# Expression of the Human R163C-*RYR1* Gain-of-Function Mutation Modified 2,2′,3,5′,6-Pentachlorobiphenyl (PCB 95) Developmental Neurotoxicity in Weanling Mice

**DOI:** 10.3390/ijms27177801

**Published:** 2026-08-31

**Authors:** Christopher D. Barnhart, Rebecca J. Wilson, Sunjay Sethi, Hao Chen, Kim M. Truong, Izabela Kania-Korwel, Hans-Joachim Lehmler, Isaac N. Pessah, Pamela J. Lein

**Affiliations:** 1 Department of Molecular Biosciences, School of Veterinary Medicine, University of California Davis, 1089 Veterinary Medicine Drive, Davis, CA 95616, USArjwilso@ucdavis.edu (R.J.W.);; 2 Department of Occupational and Environmental Health, College of Public Health, University of Iowa, Iowa City, IA 52242, USAhans-joachim-lehmler@uiowa.edu (H.-J.L.)

**Keywords:** calcium-dependent signaling, neural exposome, neurodevelopmental disorders, neuronal connectivity, polychlorinated biphenyls, ryanodine receptor

## Abstract

Epidemiological studies have identified the developing brain as a target of concern for polychlorinated biphenyls (PCBs). In animal models, behavioral deficits caused by developmental exposure to PCBs have been associated with altered patterns of dendritic arborization in functionally relevant brain regions. In vitro studies revealed that PCB 95 promoted dendritic growth in primary rat hippocampal neuron–glia co-cultures via ryanodine receptor 1 (RYR1)-dependent Ca^2+^ signaling. However, it is not yet known whether RYR1 dysregulation contributes to disruption of dendritic morphogenesis in the intact developing brain or whether PCB 95 affects translationally relevant behavioral endpoints in juvenile animals. To address these data gaps, we assessed Morris water maze (MWM) performance and dendritic arborization of hippocampal CA1 pyramidal neurons in C57BL/6 mice heterozygous for the human R163C-*RYR1* gain-of-function mutation (HET) and congenic wildtype (WT) littermates exposed to vehicle or PCB 95 at 0.1, 1.0, or 6.0 mg/kg/d in the dam’s diet from conception through weaning. MWM performance was not altered in HET vehicle controls relative to WT vehicle controls; however, compared to genotype-matched vehicle controls, spatial learning was impaired in WT and HET male and female weanlings in the 1.0 mg/kg/d PCB 95 dose group and WT males in the 6.0 mg/kg/d PCB 95 dose group. Spatial memory was altered only in WT females exposed to 1.0 or 6.0 mg/kg/d PCB 95. Sholl analyses of Golgi-stained hippocampal neurons in male and female WT and HET weanlings from the 1.0 mg/kg/d PCB 95 and vehicle groups indicated that relative to WT vehicle controls, basal dendritic arborization was significantly increased in HET vehicle controls and in PCB 95-exposed WT weanlings; however, PCB 95 did not alter basal dendritic growth in HET weanlings relative to genotype-matched vehicle controls. PCB 95 significantly reduced training-induced dendritic arborization in WT but not HET weanlings. Measurement of tritiated ryanodine ([^3^H]Ry) binding in cortical tissue from these same animals revealed interactions between genotype, PCB 95 exposure, and dose that altered [*^3^*H]Ry binding relative to WT vehicle controls. Quantitative analyses confirmed a dose-dependent increase in PCB tissue burden that was not significantly altered by genotype, MWM training, or sex. Serum levels of progesterone, estradiol, cortisol, and thyroid hormone (TH) and brain transcript levels of TH-responsive genes were not significantly altered by genotype or PCB 95 dose. These data demonstrate that PCB 95 caused behavioral deficits coincident with altered patterns of basal and training-induced dendritic arborization. Furthermore, behavioral and dendritic responses to PCB 95 were altered by expression of the human R163C-*RYR1* gain-of-function mutation, supporting the involvement of RYR1-dependent mechanisms in PCB 95 DNT in vivo.

## 1. Introduction

Behavior is specified by neural circuits [1,2], which are defined by the pattern of neuronal connections established during development [3]. A significant determinant of neuronal connectivity in the developing brain is the cytoarchitecture of the dendritic arbor, which comprises the primary site of synaptic input [4,5]. Perturbations of the spatiotemporal patterning and/or magnitude of dendritic structure have been causally linked to behavioral deficits in animal models [2,6] and are thought to be the biological basis of behavioral changes associated with human neurodevelopmental disorders [7,8,9,10,11,12,13].

Dendritic morphogenesis is regulated by the precise interplay between extrinsic molecular signals (e.g., neurotrophic factors, adhesive guidance cues, and endocrine signals) and intrinsic genetic factors (e.g., sex-dependent genetic expression, epigenetic changes in gene expression, and timing of gene expression), with further modification or refinement of the dendritic arbor by neural activity, also referred to as experience [14,15,16,17]. Experience triggers dendritic growth via ryanodine receptor (RYR)-dependent changes in calcium (Ca^2+^) signaling [18,19,20,21,22,23]. RYRs are microsomal Ca^2+^ channels broadly expressed throughout the mammalian brain that associate with cytosolic, endoplasmic reticulum (ER)-anchored, and ER luminal proteins to regulate Ca^2+^ release from the ER and modify gating responses of plasma membrane calcium channels, notably NMDA receptors and voltage-gated Ca^2+^ channels [24,25,26,27].

We previously reported that RYR-dependent Ca^2+^ signaling pathways that link neural activity to structural changes in dendritic arborization can be modulated by polychlorinated biphenyls (PCBs) [28,29,30,31]. PCBs are a class of anthropogenic persistent organic pollutants classified based on molecular structure as dioxin-like (DL) or non-dioxin-like (NDL) [32]. Although commercial production of PCBs has been banned globally since the early 2000s, NDL PCBs persist in contemporary environmental samples and human tissues [33]. Epidemiological studies have linked developmental exposure to NDL PCBs to altered neuropsychological function in infancy or childhood, and NDL PCBs have been associated with increased risk for some neurodevelopmental disorders [34,35,36,37,38,39].

We previously demonstrated that developmental exposure to Aroclor 1254 (A1254), a commercial PCB mixture that predominantly comprises NDL PCBs [33], promoted basal dendritic arborization and interfered with activity-induced dendritic growth in weanling rats, and these morphogenic effects coincided with impaired performance in the Morris water maze (MWM), a well-established test of spatial learning and memory [40]. Subsequently, we showed that PCB 95, one of the NDL congeners present in A1254, stimulated dendritic outgrowth in vivo and in vitro [30]. In primary cultures of rat hippocampal neuron–glia co-cultures, the dendrite-promoting activity of nanomolar concentrations of PCB 95 was mediated by activation of Ca^2+^-dependent signaling pathways and was inhibited by pharmacological or gene editing strategies to block RYR1 activity [29,30].

However, it is not yet known whether RYR-dependent mechanisms mediate the effects of PCBs on dendritic morphogenesis in the intact developing brain or whether PCB 95 affects translationally relevant cognitive endpoints in juvenile animals. Evaluating the contribution of RYR to in vivo PCB developmental neurotoxicity (DNT) has been challenging because RYR mutant or knockout models are embryo-lethal and genetic or pharmacologic manipulation of RYR1 or RYR2 can cause skeletal muscle or cardiac toxicity [41,42]. Here, we employed an alternative strategy, using a transgenic mouse that expresses the human R163C-*RYR1* gain-of-function mutation [43]. It is estimated that approximately one-third of the human population carries a *RYR1* variant [44,45]. Many of these variants lack phenotypic penetrance unless the individual is subjected to pharmacological triggering chemicals or environmental stressors [45]. Mechanistic studies indicate that many of these mutations result in intracellular calcium dysregulation [45]. The R163C-*RYR1* mutation sensitizes RYR1 to endogenous ligands, such as Ca^2+^ [46], and exogenous agonists, including PCB 95 [47]. Thus, if RYR1-dependent mechanisms are important in PCB 95 DNT, we predict that expression of the R163C-*RYR1* mutation will alter PCB 95 effects on dendritic arborization and MWM performance.

## 2. Results

### 2.1. Developmental Exposure to PCB 95 Altered Pup Weight in a Dose- and Sex-Dependent Manner but Did Not Cause Overt Maternal Toxicity

**Figure 1:**
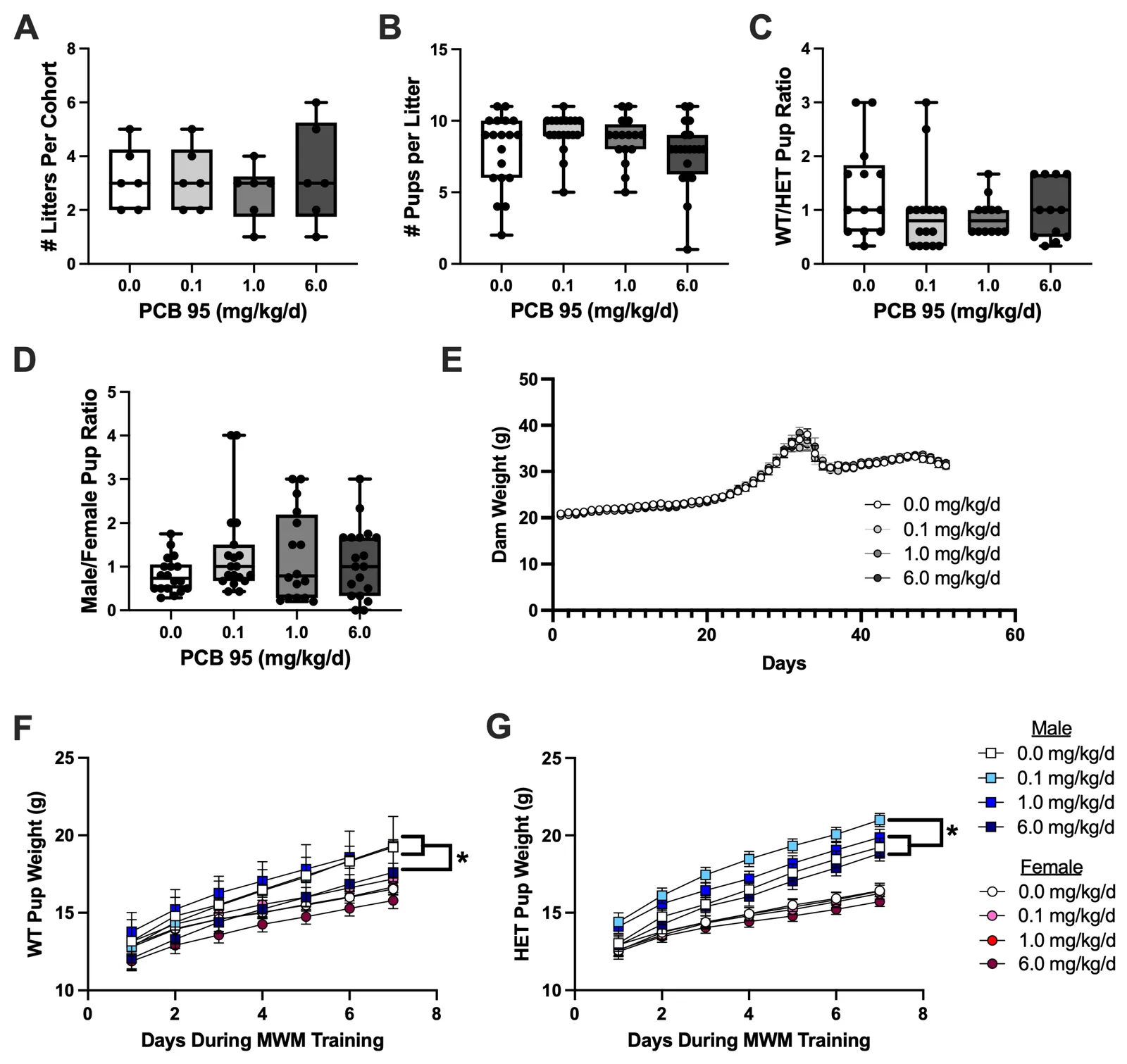
PCB 95 exposure altered pup weight in a dose- and sex-dependent manner but did not cause overt maternal toxicity. Maternal and pup toxicity was assessed as: (**A**) the number of litters born per cohort (*n* = 5 cohorts per group); (**B**) the number of pups per litter (*n* = 13–16 litters per group); (**C**) the ratio of males to females per litter (*n* = 13–16 litters per group); (**D**) the ratio of WT weanlings to HET weanlings per litter (*n* = 13–16 litters per group); (**E**) dam weight throughout the exposure period (*n* = 13–16 dams per group); and the weight of male (blue) and female (red) (**F**) WT; and (**G**) HET weanlings during MWM training (13–17 pups per genotype per group). Data presented as the mean ± SEM. * Significantly different at *p* < 0.05 as determined by linear mixed-effects model analysis.

Consistent with previous studies in rats [30,48,49] and mice [50,51], dietary exposure of dams to PCB 95 starting 2 weeks before conception and continuing until weaning at P21 had no significant effects on the number of litters per cohort (Figure 1A and Table 1), litter size (Figure 1B), or sex ratio within litters (Figure 1D). There were also no significant group differences in the ratio of WT to HET pups within litters (Figure 1C) or dam weight throughout the dosing period (Figure 1E). There was a significant effect of age on pup weight in all dose groups for both WT and HET weanlings (Figure 1F,G), indicating that pups gained weight over time regardless of PCB 95 exposure. As expected, male pups weighed significantly more than female pups in all groups. Pairwise contrasts revealed that WT pups in the 6.0 mg/kg/d dose group weighed less than the vehicle controls throughout training (males, 95% CI: −1.324 to −0.204; females, −0.998 to −0.008), whereas HET pups in the 0.1 mg/kg/d dose group weighed significantly more than the vehicle controls beginning on training day 2 and continuing through the end of the training period (training day 6; 95% CI: 0.031 to 0.997 and 0.382 to 1.341).

### 2.2. Spatial Learning Was Impaired by Developmental PCB 95 Exposure but Not RYR1 Genotype

**Figure 2:**
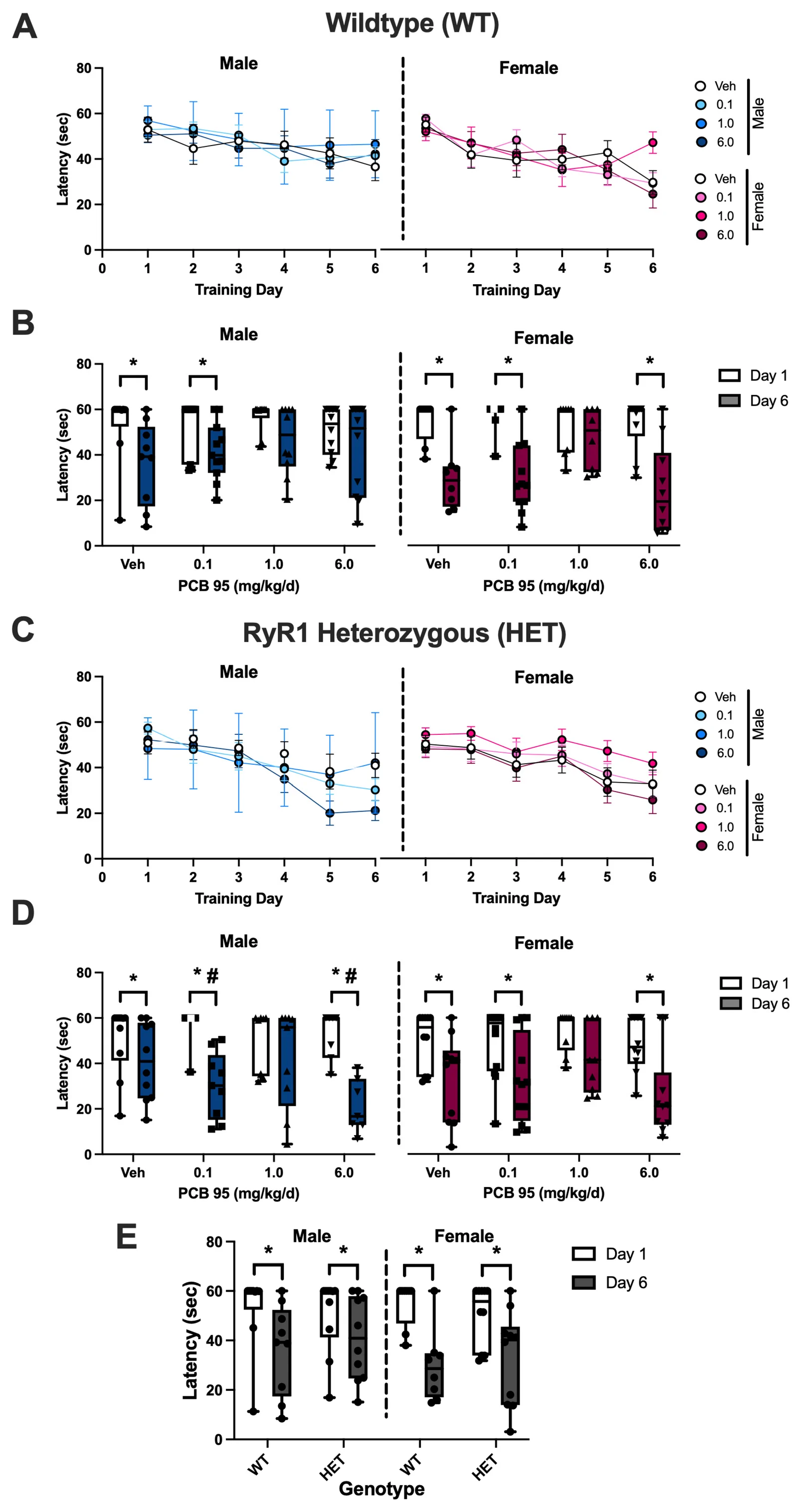
Developmental PCB 95 exposure nonmonotonically impaired spatial learning in the Morris water maze (MWM), and the magnitude of the impairment was influenced by genotype. Spatial learning was assessed using the MWM beginning at P23–25. WT (**A**,**B**) and HET (**C**,**D**) weanlings were assessed for (**A**,**C**) escape latency over the 6 d training period, (**B**,**D**) change in latency between day 1 and day 6, and (**E**) effect of genotype on escape latency as measured by the change in latency between day 1 and day 6 for WT and HET vehicle controls. Data are presented as box and whisker plots in which the box indicates the 25th (lower) to 75th (upper) quartiles; the middle horizontal bar, the median; whiskers, the minimum and maximum values; and dots, the individual values (*n* = 9–11 animals per group). * Day 6 values are significantly different from the sex-, dose-, and genotype-matched day 1 values at *p* < 0.05, # significantly different from the dose- and sex-matched WT control group at *p* < 0.05, as determined by linear mixed-effects model analysis.

We previously described a protocol for assessing MWM performance of weanling mice [52]. This protocol was used to evaluate PCB 95 dose, sex, and genotype influences on spatial learning and memory. Spatial learning, which was assessed during the training period, was assessed as: (1) latency to reach the platform and (2) the percentage of the swim path length located in the target quadrant containing the escape platform. A lack of improvement in these parameters from training day 1 to 6, the final day of the training period, suggests spatial learning impairment [52]. In WT mice exposed to PCB 95, latency to reach the platform did not improve over the training period (Figure 2A,B; F_(3,138)_ = 2.982, *p* = 0.034). Specifically, WT male pups exposed to 1.0 and 6.0 mg/kg/d PCB 95 and female pups exposed to 1.0 mg/kg/d PCB 95 did not show a significant difference in latency on day 6 of training compared to day 1 of training (Figure 2B; *n* = 9–11, *p* > 0.05). Exposure to PCB 95 did not significantly alter the percentage of swim path length spent in the target quadrant on day 6 in any dose group relative to sex-matched vehicle controls (Supplementary Figure S1). Overall, females showed a significantly decreased latency compared to males on day 6 regardless of dose group (F_(3,136.4)_ = 4.16, *p* = 0.043).

To determine whether expression of the human R163C-*RYR1* gain-of-function mutation altered spatial learning, we compared WT to HET vehicle controls. The vehicle control groups for both genotypes showed a significant decrease in latency to find the platform on day 6 compared to day 1 (Figure 2E). Pairwise comparisons indicated no significant difference between genotypes, suggesting that expression of the R163C-*RYR1* mutation alone did not impair spatial learning in the MWM task.

PCB 95 impaired spatial learning in HET male and female weanlings in the 1.0 mg/kg/d dose group as evidenced by the observation that latency on day 6 of training was not significantly different from latency on day 1 of training (Figure 2A–D; *n* = 9–11 pups per group, *p* > 0.05). Interestingly, male HET weanlings exposed to 0.1 and 6.0 mg/kg/d had a significantly larger decrease in latency between training days 1 and 6 compared to sex- and dose-matched WT weanlings (Figure 2B,D; F_(1,65.18)_ = 5.554, *p* = 0.021; F_(1,65.85)_ = 5.45, *p* = 0.023). Although PCB 95 exposure did not significantly alter the percentage of path length spent in the target quadrant within either genotype relative to genotype- and sex-matched vehicle controls, the magnitude of the PCB 95-associated change relative to vehicle control differed significantly between genotypes. Specifically, female WT weanlings exposed to 0.1 or 1.0 mg/kg/d PCB 95 exhibited a significantly greater increase in the percentage of path length spent in the target quadrant relative to genotype-matched vehicle controls compared with female HET weanlings exposed to the corresponding dose (F_(1,147)_ = 8.681, *p* = 0.004) (Supplementary Figure S1).

### 2.3. PCB 95 Effects on Spatial Memory Varied Depending on Dose and Sex and Were Modified by Genotype

**Figure 3:**
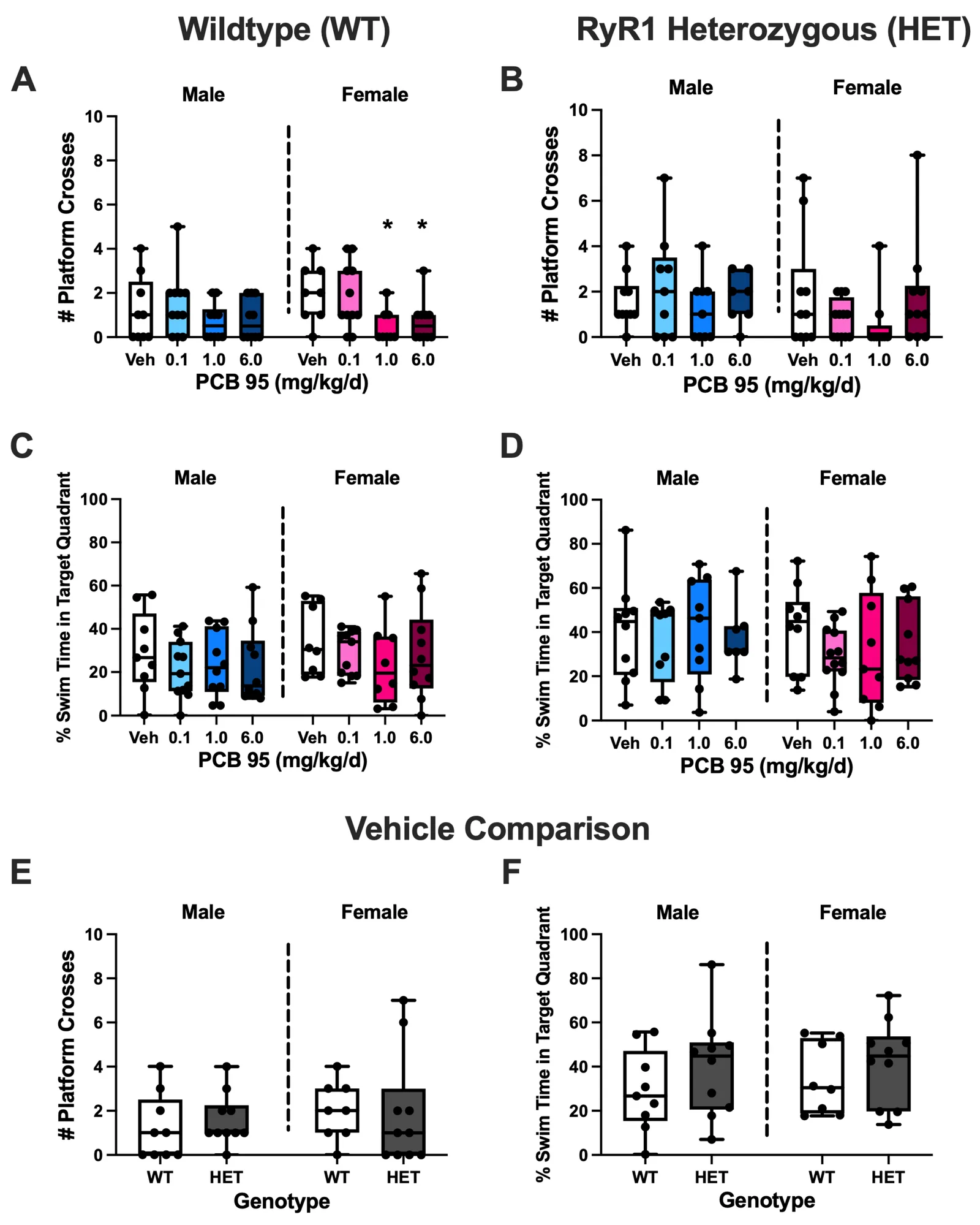
Developmental PCB 95 exposure impaired spatial memory in a sex-, dose-, and genotype-dependent manner. A probe test was administered on training day 7 to assess spatial memory in WT (**A**,**C**) and HET (**B**,**D**) weanlings. In the probe test, the submerged platform was removed, and the number of times weanling mice crossed the former platform location (**A**,**B**) and the percentage of time spent in the target quadrant (**C**,**D**) were recorded. To address genotype effects on overall MWM performance, the number of crossings over the platform location (**E**) and the percentage of time spent in the target quadrant (**F**) were compared between WT and HET vehicle controls. Data are presented as box and whisker plots in which the box indicates the 25th (lower) to 75th (upper) quartiles; the middle horizontal bar, the median; whiskers, the minimum and maximum values; and dots, the individual values (*n* = 9–11 animals per group). * Significantly different from the sex- and genotype-matched vehicle control group at *p* < 0.05 as determined by linear mixed-effects model analysis.

To assess spatial memory, a probe test was administered on training day 7 in which the platform was removed. The outcome measures included the number of crossings over the platform location during training and the percentage of time spent swimming in the target quadrant. Pairwise comparisons identified significantly reduced platform crossings in female WT weanlings exposed to PCB 95 at 1.0 and 6.0 mg/kg/d compared to sex-matched WT vehicle controls (Figure 3A;: F_(1,69)_ = 2.521, *p* = 0.014; F_(1,69)_ = 2.303, *p* = 0.025, respectively). Platform crossings were not altered in male WT weanlings exposed to PCB 95. The percent swim time spent in the target quadrant was not altered in WT females or males in any dose group (Figure 3C).

To determine whether genotype alone altered spatial memory, we compared the performance of WT vs. HET vehicle controls (Figure 3E,F). The HET vehicle control weanlings showed no significant differences in performance in the probe trial compared to WT weanlings as determined by either the number of platform crossings or the percentage of time spent swimming in the target quadrant.

To determine whether genotype-modified PCB 95 affects spatial memory, we compared the performance of HET weanlings in the probe test to that of sex- and dose-matched WT weanlings. In the PCB-exposed HET weanlings, the number of platform crossings was not significantly different compared to vehicle controls (Figure 3B). As observed for the WT weanlings, PCB 95 exposure did not significantly alter the percentage of time spent in the target quadrant for male or female HET weanlings (Figure 3D). The decrease in percent time PCB 95-exposed weanlings spent in the target quadrant relative to genotype-matched vehicle controls was significantly less in HET weanlings compared to WT weanlings (F_(1,76)_ = 4.599, *p* = 0.035; F_(1,69)_ = 5.802, *p* = 0.019; F_(1,65.49)_ = 5.213, *p* = 0.026, respectively).

To rule out the possibility that MWM performance was impaired because exposure to PCB 95 caused deficits in sensorimotor function or motivation, a visual cue test was administered after the probe trial. Mean swim velocity (Supplementary Figure S2A,B) and escape latency in the visual cue test (Supplementary Figure S2C,D) were not significantly affected by PCB 95 dose, sex, or genotype.

### 2.4. PCB 95 Effects on Basal and Training-Induced Dendritic Arborization Were Altered by RYR1 Genotype

Performance in the MWM is mediated by neural circuits in the hippocampus, neocortex, and cerebellum [53], and within the hippocampus, the CA1 region of the hippocampus is particularly important for the formation of spatial memory in MWM training [54]. Therefore, to determine whether morphometric indices of neuronal connectivity were altered by developmental PCB 95 exposure, R163C-*RYR1* expression, MWM training, or sex, the basilar arbors of Golgi-stained CA1 hippocampal pyramidal neurons were quantified using Sholl analysis in weanlings that had completed MWM training and in sex-, age-, and genotype-matched littermates that had not undergone MWM training. This study focused on neurons from weanlings exposed to vehicle or PCB 95 at 1.0 mg/kg/d since both WT and HET weanlings exposed to this dose of PCB 95 exhibited significant deficits in spatial learning and memory compared to vehicle controls (Figure 2 and Figure 3). There were no significant differences between male and female weanlings, so data were pooled (Supplementary Figure S3; WT: F_(3,20)_ = 0.0688, *p* = 0.9759; HET: F_(3,20)_ = 0.7815, *p* = 0.5182).

**Figure 4:**
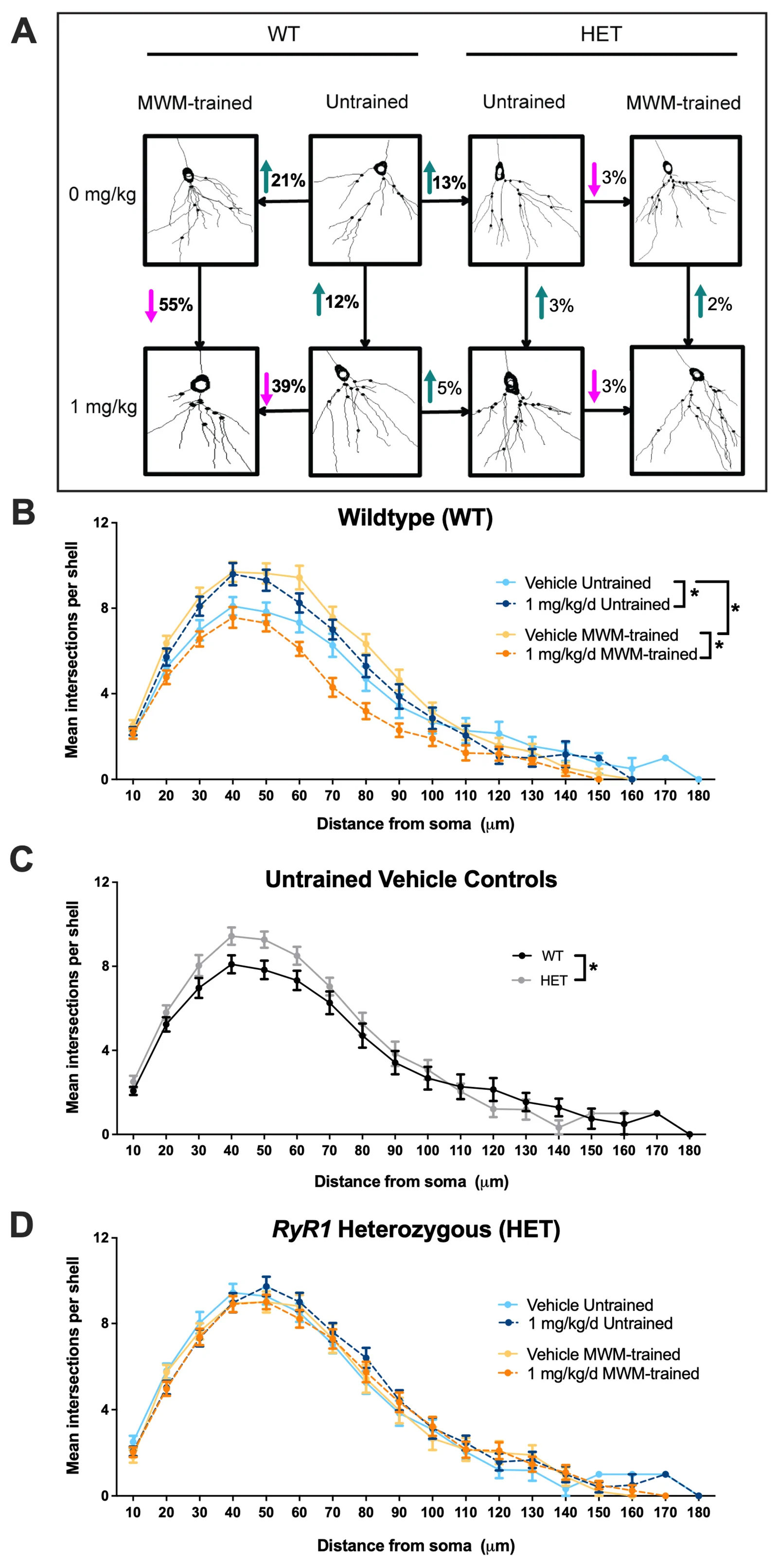
PCB 95, MWM training, and R163C-*RYR1* expression modified dendritic arborization. (**A**) Representative tracings of the basilar dendritic arbors of Golgi-stained CA1 hippocampal pyramidal neurons. Percentages indicate the difference in the average total number of Sholl intersections per animal between experimental groups, expressed as the percent change from the comparison group at the origin of the black arrow to the group at the arrowhead with up arrows indicating an increase and down arrows indicating a decrease. (**B**–**D**) Sholl plots were generated from Sholl analysis of the basilar arbors of CA1 pyramidal neurons in (**B**) trained and untrained WT weanlings exposed to vehicle vs. PCB 95 at 1.0 mg/kg/d in the maternal diet; (**C**) untrained WT vs. HET vehicle controls; and (**D**) trained and untrained HET weanlings exposed to vehicle vs. PCB 95 at 1.0 mg/kg/d in the maternal diet. Data are presented as the mean ± SEM (*n* = 5 animals per experimental condition, with males and females pooled; the value obtained for each individual animal represents the mean of 6–7 neurons). * Significantly different at *p* < 0.05 as determined by a linear mixed-effects model.

There was a main effect of PCB 95 on dendritic morphology in untrained WT weanlings as determined using a linear mixed-effects model (Figure 4A,B; F_(3,20)_ = 6.741; *p* = 0.0025). Specifically, dendritic arborization was significantly increased in PCB 95-exposed WT weanlings relative to untrained WT vehicle controls (Figure 4A,B; 95% CI = 0.50, 2.86, *p* = 0.007). Similarly, MWM training significantly increased dendritic arbor complexity in WT vehicle controls (Figure 4A,B; 95% CI = 3.615, 14.85, *p* = 0.0211). However, exposure to PCB 95 at 1.0 mg/kg/d reversed the effects of MWM training on dendritic growth in WTs, as evidenced by a significant decrease in the mean number of intersections per shell per neuron compared to WT vehicle controls with MWM training (Figure 4A,B).

Heterozygous expression of R163C-*RYR1* increased dendritic arborization of CA1 pyramidal neurons in untrained weanlings compared to untrained WT vehicle controls, as evidenced by a significant increase in the mean ratio of the total Sholl intersections per neuron in HETs versus WTs (Figure 4A,C; 95% CI = 14%, 78%, *p* = 0.002). There was also a significant 1.65-fold increase in the mean area under the curve (AUC) of smoothed neuron-specific Sholl profiles of untrained HETs versus untrained WT controls (Figure 4C; 95% CI = 0.67, 2.63, *p* = 0.0012). In contrast to WT weanlings, neither MWM training nor exposure to PCB 95 at 1.0 mg/kg/d significantly altered dendritic arborization in CA1 neurons of HET weanlings (Figure 4D; F_(3,20)_ = 0.1864, *p* = 0.1864). There were also no significant differences in dendritic complexity between trained WTs and trained HETs as determined by the mean ratio of the area under the Sholl plot (95% CI = −25%, 22%, *p* = 0.9982) or mean difference in AUC (95% CI = −1.52, 1.03, *p* = 0.9419).

### 2.5. PCB Tissue Burdens Increased with Increasing Dose

**Figure 5:**
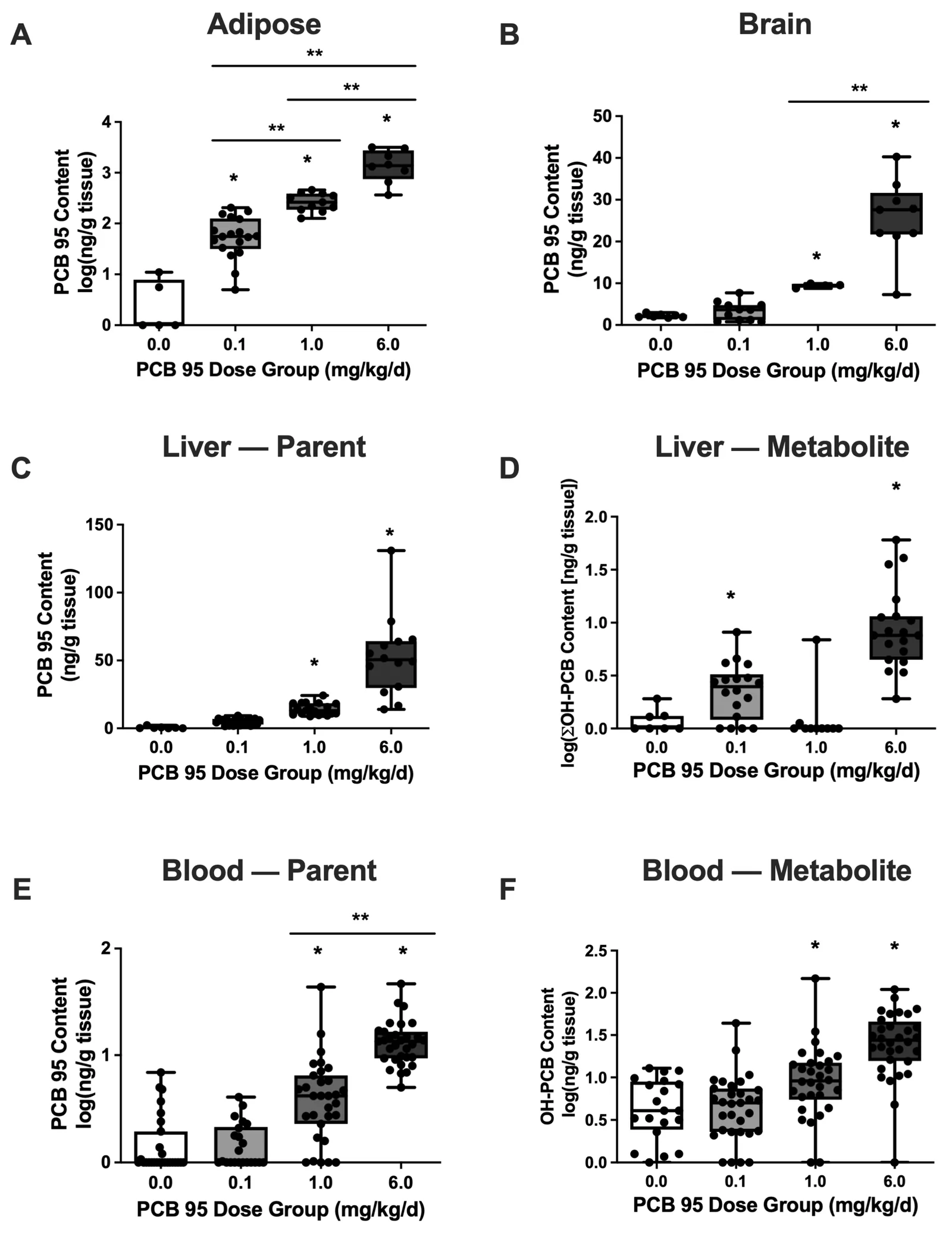
Tissue levels of PCB 95 parent compound and metabolites increased with increasing dose. Samples of adipose (**A**), brain (**B**), liver (**C**,**D**) and blood (**E**,**F**) were collected at P31–33 for analysis of the parent compound, PCB 95 (**A**–**C**,**E**), and its hydroxylated metabolites (OH-PCBs; (**D**,**F**)). Of these tissues, only the liver and blood contained detectable levels of OH-PCBs. Data are presented as box and whisker plots in which the box indicates the 25th (lower) to 75th (upper) quartiles; the middle horizontal bar, the median; whiskers, the minimum and maximum values; and dots, individual animal values (*n* = 5–19 animals per group); data were collapsed across sex, genotype, and training. * Significantly different from the vehicle control group at *p* < 0.05, ** significantly different from the specified dose group at *p* < 0.05 as determined by one-way ANOVA with *post hoc* Tukey’s test. Levels of PCB 95 and its metabolites in the control samples represent the background signal for the respective analyte in the GC-ECD analysis.

To determine whether genotype differences in PCB effects on performance in the MWM and/or dendritic outcome measures were related to genotype effects on tissue distribution and/or metabolism of PCB 95, tissue burdens of PCB 95 and five of its hydroxylated metabolites were quantified in adipose, brain, liver, and blood. PCB 95 content was significantly higher in adipose relative to the other tissues analyzed and decreased in the rank order adipose > liver > brain > blood (Figure 5). Across all dose groups, levels of PCB 95 in adipose tissue were 5 to 20 times higher than in the liver, 25 to 40 times higher than in the brain, and 35 to 100 times higher than in the blood. There were no significant effects of genotype, sex, or training on levels of the PCB 95 parent compound or metabolites in any of these tissues, so these samples were pooled. PCB 95 burdens increased proportionally with dose in all tissues (Figure 5A–C and E, respectively). Briefly, there were significant main effects of dose on PCB 95 levels in adipose (log scale, F_(3,38)_ = 67.70, *p* < 0.0001), brain (F_(3,27)_ = 38.45, *p* < 0.0001), liver (F_(3,60)_ = 40.02, *p* < 0.0001), and blood (log scale, F_(3,107)_ = 74.95, *p* < 0.0001), as determined by one-way ANOVA. More specifically, in adipose tissue, the animals exposed to 0.1, 1.0, and 6.0 mg/kg/d PCB 95 had significantly higher PCB 95 content compared to vehicle controls (Figure 5A; log scale, 95% CI = −1.859, −0.8696, *p* < 0.0001; 95% CI = −2.588, −1.532, *p* < 0.0001; 95% CI = −3.326, −2.210, *p* < 0.0001, respectively). In brain and liver tissue, the animals exposed to 1.0 and 6.0 mg/kg/d PCB 95 had significantly higher PCB 95 content (Figure 5B; 95% CI = −28.85, −16.00, *p* < 0.0001; 95% CI = −69.26, −35.14, *p* < 0.0001; Figure 5C; 95% CI = −60.12, −35.34, *p* < 0.0001; 95% CI = −30.76, −16.35, *p* < 0.0001, respectively). Finally, in blood samples, the animals exposed to 1.0 and 6.0 mg/kg/d PCB 95 showed significantly higher PCB 95 content (Figure 5E; 95% CI = −0.6145, −0.2321, *p* < 0.0001; 95% CI = −1.148, −0.7653, *p* < 0.0001, respectively).

Five hydroxylated PCB 95 metabolites (3-103, 4-95, 4′-95, 5-95, and 4,5-95) were also measured in these four tissues (Figure 5). There were no effects of sex, genotype, or training, so these samples were pooled. In the liver, there were main effects of dose on the sum of all PCB metabolites (Figure 5D). There were significantly more OH-PCBs in liver samples, primarily 4-95 and 5-95, collected from pups exposed to 0.1 and 6.0 mg/kg/d PCB 95 compared to vehicle controls (95% CI = −0.8483, −0.3216, *p* < 0.0001; 95% CI = −1.122, −0.5137, *p* < 0.0001, respectively). The 4-95 hydroxylated metabolite was the major metabolite detected in the blood, with 4-95 levels being higher than PCB 95 levels in the 6.0 mg/kg dose group. PCB 95 metabolite tissue burdens in the blood increased with dose in the rank order 6.0 mg/kg/d > 1.0 mg/kg/d > 0.1 mg/kg/d > vehicle control (Figure 5F). There were significant main effects of dose (F_3,94.1_ = 27.931, *p* < 0.0001) (Figure 5F). Levels of PCB 95 metabolites in adipose and brain tissue were below the detection limit in all groups, which has been observed previously [51]. Other PCB 95 metabolites may have been present in serum or the tissues investigated, but they were not quantified due to the unavailability of analytical standards.

### 2.6. PCB 95 Had Negligible Effect on Thyroid and Steroid Hormone Levels or Thyroid Hormone-Responsive Gene Expression

Developmental PCB exposure has been linked to alterations in thyroid hormones and steroid hormones important for development [55,56]. To assess endocrine disruption as a potential mechanism contributing to PCB 95 DNT in vivo, serum concentrations of T3, T4, progesterone, estradiol, and cortisol were measured at P31–33 in WT and HET animals [52]. There were no main effects of PCB 95 exposure or R163C-*RYR1* expression on any of the measured circulating hormones, but there were effects of sex and training (Supplementary Tables S1 and S2). Specifically, there were significant effects of training and sex on cortisol levels. MWM-trained animals exposed to 0.1, 1.0, and 6.0 mg/kg/d PCB 95 had significantly higher cortisol than untrained animals. Additionally, females exposed to 0.1, 1.0, and 6.0 mg/kg/d PCB 95 had significantly higher cortisol than males overall. There were also significant effects of training and sex on progesterone levels. MWM-trained animals exposed to 0.1 mg/kg/d PCB 95 showed significantly higher progesterone levels than their untrained counterparts, and female animals exposed to 6.0 mg/kg/d PCB 95 showed significantly lower levels of progesterone than their male counterparts. Training also significantly altered serum levels of T3, and sex influenced serum levels of T4. Regarding T3 levels, MWM-trained animals exposed to 0.1 mg/kg/d PCB 95 had significantly lower T3 levels compared to untrained controls. Female weanlings exposed to 0.1 mg/kg/d PCB 95 had significantly higher T4 levels than male weanlings.

PCBs and their metabolites can also disrupt endocrine system function by interacting with the TH receptor [57] to modulate expression of TH-responsive genes [58]. To address this possibility, we quantified transcript levels of the TH-responsive genes myelin basic protein and neurogranin in hippocampi, cortices, and cerebella collected from weanlings 24 h after the conclusion of MWM training [52]. The only dose- and genotype-dependent modulation of TH-responsive gene expression was observed in the hippocampi of MWM-trained weanlings in the 0.1 and 6.0 mg/kg/d dose groups (Supplementary Tables S3–S5).

### 2.7. Cortical RYR Channel Configuration Is Altered by R163C-RYR1 Expression, PCB Exposure, and MWM Training

**Figure 6:**
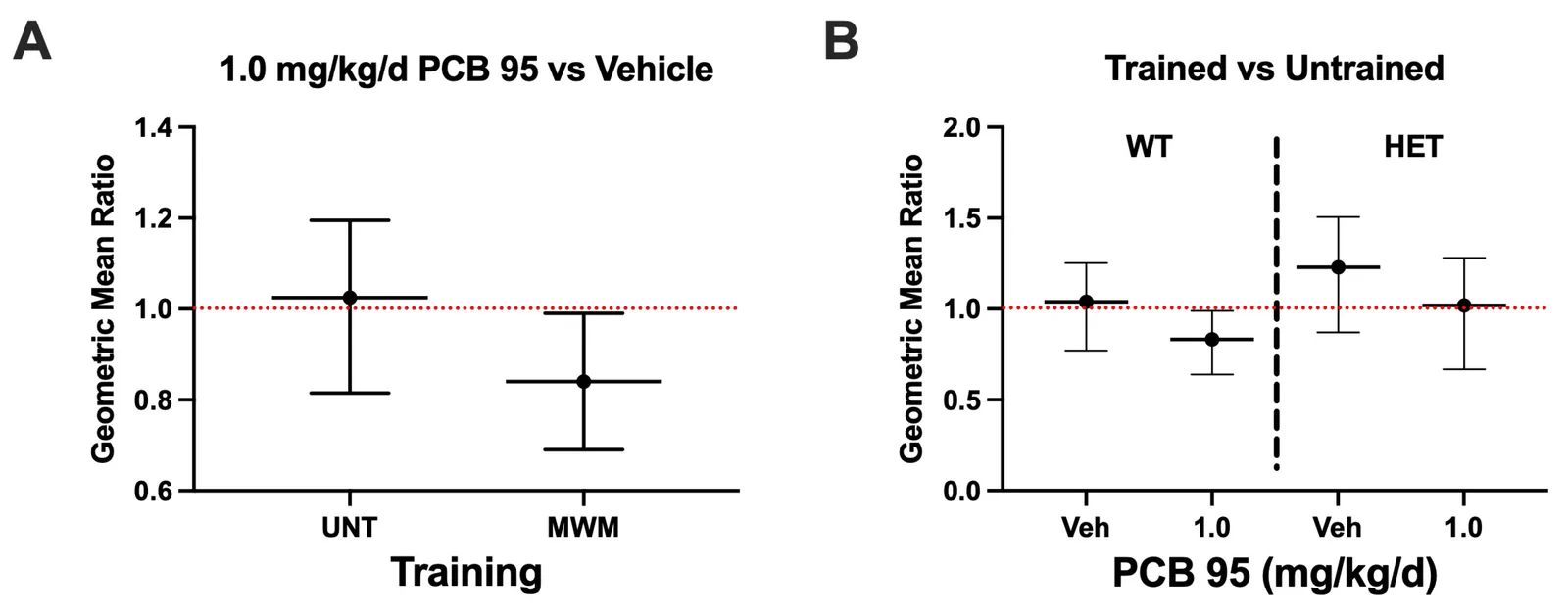
Exposure to 1.0 mg/kg/d PCB 95 and Morris water maze training affected tritiated ryanodine binding in the cortex. Levels of bound [^3^H]Ry were measured in cortical microsomes from weanlings at P31–33. Samples from males and females were pooled. (**A**) To evaluate the effect of PCB exposure on RYR, [^3^H]Ry binding was compared in PCB 95-exposed vs. vehicle controls; untrained (UNT) and trained (MWM) littermates were evaluated separately (samples were pooled across genotype). (**B**) To evaluate the effect of training on RYR, [^3^H]Ry binding was compared in trained vs. untrained controls; genotype and dose groups were evaluated separately. Data are presented as the geometric mean ratio (dot) of the two groups being compared and 95% confidence intervals (bars) (*n* = 6–9 membrane preparations per dose group). Confidence intervals that do not include 1 (dotted line) indicate a significant difference between groups.

PCB 95 [59] and expression of the R163C-*RYR1* gain-of-function mutation [46,47] have previously been shown to stabilize RYR channels in their open configuration, which increases release of Ca^2+^ from the ER into the cytoplasm to activate Ca^2+^-dependent signaling pathways, including the signaling pathway that links PCB 95 and bicuculline-induced neural activity to dendritic growth [29]. The RYR ligand, ryanodine, only binds to the channel in its open configuration [25,59], so [^3^H]Ry binding assays were performed on cortical microsomal preparations from the same weanlings used for the Golgi stain analyses to determine whether exposure to PCB 95 at 1.0 mg/kg/d (Figure 6A) and/or MWM training (Figure 6B) altered the number of open RYR channels. We used cortical tissue for these studies because it yields more material for biochemical studies than the hippocampus, but like the hippocampus, neural circuits in the neocortex are important for MWM performance [53]. There was no significant effect of sex on this outcome measure, so samples from males and females were pooled. As determined by assessing geometric mean ratios ± 95% confidence intervals to compare data from PCB 95 at 1.0 mg/kg/d vs. vehicle on [^3^H]Ry binding in cortices pooled from WT and HET weanlings (Figure 6A), PCB 95 had no significant effect on RYR channel configuration in untrained WT weanlings, but significantly decreased [^3^H]Ry binding in MWM-trained animals (Figure 6A; GMR = 0.84; 95% CI: 0.69–0.96, *p* = 0.039). Comparison of the effect of training in the absence or presence of PCB 95 revealed differences between genotypes. In WT animals, training had no effect on [^3^H]Ry binding in vehicle controls but significantly decreased the number of open RYR channels in PCB 95-exposed animals (GMR = 0.83; 95% CI: 0.68–0.98, *p* = 0.046). In contrast, training increased the number of open RYR channels in HET vehicle controls, although this difference did not reach statistical significance (GMR = 1.23; 95% CI: 0.87–1.50, *p* = 0.057) but had no effect on this measure in PCB 95-exposed weanlings (Figure 6B).

## 3. Discussion

Previous studies demonstrated that PCB 95, an NDL congener with potent RYR activity [59], promoted dendritic growth of pyramidal neurons in the developing rat hippocampus. Mechanistic studies found that PCB 95 promoted dendritic growth in primary rat hippocampal neuron–glia co-cultures via RYR-dependent mechanisms [30]. The findings of this study extend these earlier findings to provide evidence suggesting that RYR-dependent mechanisms are involved in PCB 95 DNT in vivo. Several observations support this conclusion.

First, in weanling WT mice of both sexes, developmental exposure to PCB 95 at 1.0 mg/kg/d in the dam’s diet from conception through weaning significantly enhanced basal dendritic arborization and reversed training-induced dendritic growth in hippocampal CA1 pyramidal neurons coincident with significantly impaired MWM performance. Second, PCB 95 effects on dendritic arborization and MWM performance coincided with a decrease in RYR activity in cortical tissue as indicated by significantly less [^3^H]Ry binding in trained vs. untrained WT weanlings exposed to PCB 95. Third, expression of the human R163C-*RYR1* gain-of-function mutation altered the effects of PCB 95 on these neurodevelopmental outcomes. Importantly, these neurodevelopmental outcomes were observed in the absence of significant genotype or PCB effects on several indices of general maternal and pup health, suggesting that the neurodevelopmental outcomes observed in PCB 95-exposed weanlings reflect direct neurotoxic effects and not the results of maternal toxicity. Collectively, these data also revealed that PCB DNT is influenced by complex interactions between dose, sex, and genotype.

An important observation from this study is that developmental exposure to PCB 95 significantly impaired MWM performance in weanling mice. Numerous studies have shown that developmental exposure to legacy commercial PCB mixtures or experimental mixtures of DL and NDL PCB congeners impairs cognitive function in experimental animal models (reviewed in [60]). While there is an earlier report of cognitive outcomes observed in rats exposed to PCB 95 during development [49], cognitive function was assessed in *adult* rats, and the data showed that PCB 95 enhanced some measures of learning and memory while impairing others. In contrast, our data demonstrated that developmental exposure to PCB 95 is sufficient to cause cognitive deficits in a weanling mouse model. Specifically, we observed that in WT weanling mice, spatial learning in the MWM was impaired in males exposed to PCB 95 at 1.0 or 6.0 mg/kg/d in the maternal diet and in females in the 1.0 mg/kg/d group. While there were subtle sex differences in the relationship between PCB 95 dose and deficits in spatial learning, there was a marked sex difference in PCB 95-induced deficits in spatial memory, with only WT females exposed to PCB 95 at 1.0 or 6.0 mg/kg/d in the dam’s diet showing impaired performance in the MWM probe test.

We had predicted that if RYR-dependent mechanisms are involved in PCB 95 DNT, then expression of the R163C-*RYR1* gain-of-function mutation would alter PCB 95 effects on MWM performance. Our observations are consistent with this prediction. Expression of the human R163C-*RYR1* mutation had no effect on MWM performance in the absence of PCBs. Interestingly, previous studies found that expression of the human T4826I-*RYR1* gain-of-function mutation altered social behavior in weanling mice [61]. While the biological mechanism(s) underlying this discrepancy remain to be determined, two possible explanations include differential biological effects of these two *RYR1* mutations and/or distinct roles for RYR1 in modulating cognitive vs. social behavior. However, expression of either human *RYR1* gain-of-function mutation modified the effects of developmental PCB exposures on behavioral phenotypes in a sex-dependent manner. Here, we observed that in the R163C-*RYR1* HET males, PCB 95-induced deficits in spatial learning were observed only in the 1.0 mg/kg/d dose group and not in the 6.0 mg/kg/d dose group, as was the case with the WT males. Further, the performance of HET males in the 0.1 and 6.0 mg/kg/d PCB 95 dose groups was significantly improved relative to their sex- and dose-matched WT counterparts. In female HETs, like WT females, spatial learning was impaired in the 1.0 mg/kg/d dose groups, but in contrast to the WT females, exposure to PCB 95 at any of the three doses had no effect on spatial memory in HET females. Importantly, there were no PCB 95 dose or genotype differences in performance on the MWM visual cue test, suggesting comparable motor, visual, and motivational ability across all groups. Collectively, these observations support a model in which the development of cognitive behavior is influenced by interactions between *RYR1* genotype, PCB 95 dose, and sex.

Non-linear dose-related behavioral responses to developmental PCB 95 exposure have been previously reported. For example, nonmonotonic dose-related deficits in MWM performance were observed in weanling rats exposed to the commercial PCB mixture A1254 in the maternal diet [40] and in the social behavior of weanling mice exposed to the MARBLES PCB mixture in the maternal diet [61]. Interestingly, it has also been previously reported that PCB 95 effects on dendritic arborization of pyramidal neurons in the hippocampus of weanling rats exposed to the same dose range of PCB 95 used in this study were nonlinear, with significantly enhanced basal dendritic growth observed in the hippocampus of animals exposed to PCB 95 at 0.1 or 1.0, but not 6.0 mg/kg/d in the maternal diet [30]. One limitation of this study was that the analysis of dendritic arborization focused exclusively on vehicle- and 1.0 mg/kg/d PCB 95-exposed animals, precluding assessment of whether changes in dendritic morphology paralleled the nonmonotonic behavioral dose–response observed across PCB 95 doses.

One biologically plausible mechanism that could contribute to such nonlinear dose-related effects is dose-dependent induction by PCBs of cytochrome P450 enzymes [62,63] that metabolize PCBs [63,64,65], thereby influencing concentrations of the parent congener and/or its metabolites in the brain. However, GC-ECD analyses of brain and other tissues from PCB 95-exposed weanlings confirmed linear dose-dependent levels of PCB 95 parent and metabolites, with the highest levels detected in tissues from animals exposed to the highest dose. The rank order of levels of PCB 95 and its metabolites in tissues and blood was in agreement with those detected previously in P21 rat pups exposed to PCB 95 at 1.0 mg/kg/d [50] and P30-P33 rat pups exposed to the same PCB 95 doses used in this study [51]. Importantly, there were no significant sex, genotype, or training differences in tissue levels of PCB 95 parent or metabolites, suggesting that neither the nonmonotonic dose-related effects of PCB 95 on MWM performance nor sex and genotype differences in the behavioral impacts of PCB 95 are mediated by differences in PCB 95 disposition across groups.

Another potential mechanism underlying nonmonotonic dose-related effects of PCB 95 on MWM performance is endocrine disruption, which classically exhibits nonlinear dose-related effects [66,67]. It has been postulated that disruption of signaling by thyroid hormones [68,69,70] and/or sex steroids [71,72] is causally related to PCB DNT. However, we did not observe a significant effect of PCB 95 dose or genotype on serum levels of thyroid hormones T3 and T4 in P31–33 weanlings. Additionally, qPCR analyses of hippocampal, cortical, and cerebellar tissue from these animals revealed no significant PCB 95- or genotype-related changes in transcript levels of the TH-responsive genes myelin basic protein and neurogranin. Measurement of the steroid hormones, estradiol, progesterone, and cortisol, in the serum of weanlings revealed that circulating progesterone levels were elevated in male weanlings compared to female weanlings, while cortisol levels were generally increased in female weanlings vs. male weanlings and in trained vs. untrained weanlings of both sexes. However, there were no significant effects of PCB 95 dose or genotype on the serum levels of any of these steroid hormones, and altered cortisol levels had no obvious impact on spatial cognition. While we cannot rule out the possibility that PCB 95 and/or genotype interfered with signaling by these hormones at earlier times in development, our data support the hypothesis that PCB 95 effects on MWM performance are not primarily mediated by endocrine disruption. Consistent with this conclusion, we had earlier shown that PCB 95 has neither agonistic nor antagonistic effects on thyroid hormone receptor function in vitro [73].

A second key observation from this study is that PCB 95-induced cognitive deficits coincided with significant effects of PCB 95 on the morphogenesis of basilar dendrites extended by hippocampal CA1 pyramidal neurons. Since MWM performance of all weanlings regardless of sex or genotype was impaired by PCB 95 at 1.0 mg/kg/d in the maternal diet, we compared dendritic arborization in this dose group relative to sex- and genotype-matched vehicle controls. Interestingly, while genotype alone had no effect on MWM performance, comparative analyses of WT vs. HET vehicle controls revealed that expression of the R163C-*RYR1* mutation significantly increased dendritic arborization of hippocampal CA1 pyramidal neurons in the absence of PCB exposure. This is consistent with an earlier study in which expression of the T4826I-*RYR1* gain-of-function mutation was observed to significantly enhance the dendritic arborization of hippocampal CA1 pyramidal neurons of weanling mice, although this morphogenic phenotype was only observed in male T4826I-*RYR1* mice and not in T4826I-*RYR1* females [74].

Genotype also influenced the dendritic response of hippocampal CA1 pyramidal neurons to PCB 95 exposure. In the absence of MWM training, PCB 95 significantly increased dendritic arborization in male and female WT weanlings but had no effect on dendritic arborization in male and female HET weanlings. The magnitude of dendritic arborization observed in the PCB 95-exposed WT weanlings was comparable to that observed in the HET vehicle controls, suggesting that PCB 95 phenocopied the effect of the R163C-*RYR1* mutation. The dendritic growth observed in untrained WT weanlings exposed to PCB 95 is consistent with a previous report of increased dendritic arbor complexity of pyramidal neurons in the CA1 hippocampus of Long-Evans rat weanlings exposed to PCB 95 at the same dose via a similar exposure paradigm [30]. In contrast, developmental exposure to the MARBLES PCB mixture did not significantly alter dendritic morphogenesis of hippocampal pyramidal neurons in untrained WT mice of either sex, but significantly increased dendritic arborization of cortical neurons in WT males exposed to the MARBLES mix at 6 mg/kg/d [75]. Also, in contrast to our observation in the current study of a lack of dendritic response to PCB 95 in HETs, expression of the T4826I-*RYR1* gain-of-function mutation increased the dendritic response to the MARBLES mix. The most likely explanation for the discrepant findings between these studies of exposure to PCB 95 vs. the MARBLES PCB mix is that, while PCB 95 is present in the MARBLES mix, it is the most minor constituent among the 12 congeners in the MARBLES mix, which included DL and NDL PCB congeners with varying levels of RYR activity [73].

In WT vehicle control weanlings, MWM training induced significant expansion of the basilar dendritic arbor of hippocampal CA1 pyramidal neurons, consistent with prior observations of experience-dependent dendritic growth in rodent models [53,76]. However, in PCB 95-exposed WT weanlings, MWM training caused dendritic retraction, as evidenced by significantly reduced complexity of the dendritic arbor relative to untrained PCB 95-exposed and vehicle-exposed trained WT weanlings. A similar antagonistic effect of developmental PCB exposure on activity-dependent dendritic growth was reported in weanling rats exposed to A1254 in the dam’s diet [40]. In striking contrast, MWM training had no significant effect on dendritic arborization in either vehicle or PCB 95-exposed HETs.

Collectively, data from WT weanlings support the hypothesis that PCB 95 effects on dendritic arborization in a brain region critical for spatial learning and memory [53] are related to PCB 95-induced deficits in MWM performance. The observation that PCB 95 had no significant effects on dendritic arborization in untrained or trained HET weanlings may seem inconsistent with the hypothesis that PCB 95 effects on spatial cognition are mediated by disturbances in dendritic growth and/or plasticity. However, HET vehicle controls did perform better than WT vehicle controls on one parameter of spatial learning (% swim time), and the increased dendritic arborization observed in HETs did coincide with no significant differences in spatial memory between PCB-exposed and vehicle control weanlings in the probe test. It is also important to note that MWM performance relies on not only hippocampal function but also cortical and cerebellar function [77,78,79]. While we did not measure dendritic arborization in these brain regions in this study, previous studies found that developmental exposure to A1254 at a dose that impaired MWM performance of weanling rats also interfered with basal and activity-dependent dendritic arborization in the cortex and cerebellum of these animals [40]. Another study reported that the MARBLES PCB mix, which contains PCB 95 and other RYR-active PCB congeners [73], altered basal dendritic growth in the cortex, but not hippocampus, of weanling mice [75]; activity-dependent dendritic growth was not measured in the MARBLES-exposed mice. It may also be that the stable pattern of dendritic arborization in HETs contributed to their improved spatial cognitive function relative to WT controls following exposure to PCB 95 at 0.1 and 6.0 mg/kg/d.

Dendritic arborization is influenced by thyroid hormones [80,81] and cortisol [82,83]. However, as previously discussed, we observed no significant effects of PCB 95 on the serum levels of these hormones or on transcript levels of TH-responsive genes in the brain. Rather, our data support an alternative mechanism involving PCB 95 effects on the RYR. First, we previously demonstrated that PCB 95 promoted dendritic growth in primary rat hippocampal neurons via RYR-dependent signaling [30], and here we show that developmental exposure of WT weanlings to PCB 95 elicited significant dendritic growth in hippocampal CA1 pyramidal neurons. Second, this effect was phenocopied by expression of the R163C-*RYR1* gain-of-function mutation. Third, expression of the R163C-*RYR1* mutation altered the relationship between PCB 95 dose and effects on spatial learning and memory and dendritic growth. Fourth, cortical [^3^H]Ry binding was significantly decreased in MWM-trained WT weanlings exposed to 1.0 mg/kg/d PCB 95, indicating a reduced number of open RYR channels, and this coincided with significant dendritic retraction in the hippocampus and deficits in MWM performance.

How PCB 95 promotes dendritic growth of hippocampal CA1 pyramidal neurons in vivo remains to be established, but Ca^2+^-dependent signaling is likely involved [84]. PCB 95 binds to RYR1 in hippocampal neurons to stabilize channels in their open configuration [59]. This interaction has been linked to increased dendritic arborization via activation of the calcium/calmodulin kinase-I (CaMKI)/cAMP response element binding protein (CREB)/Wnt2 signaling pathway, as demonstrated in primary hippocampal neurons in vitro [29]. The increased complexity of basal dendritic arborization associated with expression of the R163C-*RYR1* mutation may be mediated by the same signaling pathway since this mutation has been shown to increase intracellular Ca^2+^ concentrations in primary hippocampal neurons [46]. Why expression of the R163C-*RYR1* mutation does not shift the PCB 95 dose–response curve to the left or why exposure to PCB 95 does not further amplify dendritic arborization in HET weanlings remain outstanding questions. Possible explanations derive from in vitro observations that in early stages of neuronal development, intracellular Ca^2+^ enhances dendritic growth in a concentration-dependent manner [85]. However, in later stages of neuronal maturation, moderate increases in Ca^2+^ stimulate dendritic growth, whereas large increases promote dendritic retraction [85,86]. These suggest that interactions between PCB 95 and training may increase intracellular Ca^2+^ from concentrations that promote dendritic growth to those that cause dendritic retraction.

In conclusion, while our data do not establish an exclusive or direct causal relationship between PCB 95 sensitization of RYR1 and PCB 95 effects on behavior or dendritic arborization, they do support the involvement of RYR-dependent mechanisms in PCB 95 DNT in vivo. The translational relevance of these findings to humans is suggested by several factors. First, PCB 95 was detected in the serum of pregnant women at increased risk for giving birth to a child with a neurodevelopmental disorder [73], and the dose of PCB 95 we found to impair spatial learning independent of sex or genotype—1.0 mg/kg/d in the dam’s diet from conception through weaning—resulted in brain levels of PCB 95 of 12 ng/g tissue. For comparison, PCB 95 levels in the brains of children diagnosed with the neurodevelopmental disorder 15q duplication reached 1.9 ng/g tissue [87]. Second, *RYR1* was identified as a candidate ASD risk gene in a genome-wide association study [88], and RYR-active PCBs were linked to increased risk for autism spectrum disorder in the MARBLES cohort [89]. Third, altered spatiotemporal patterns of dendritic morphogenesis are considered the anatomic basis for many of the clinical phenotypes associated with various neurodevelopmental disorders [90,91,92,93,94]. In summary, this study demonstrates that developmental exposures to PCB 95, and possibly other RYR-active PCB congeners, can cause phenotypes of relevance to human neurodevelopmental disorders and identifies a complex neural exposome profile (dose, sex, training, and *RYR* genotype) that modifies individual risk for PCB DNT.

## 4. Materials and Methods

### 4.1. Animals

Animals were treated humanely with attention to the alleviation of pain and suffering in accordance with protocols approved by the Institutional Animal Care and Use Committee of the University of California, Davis (protocol code 17068, date of approval: 20 March 2015). Male C57BL/6 mice heterozygous for the R163C-RYR1 mutation (HET) were obtained from an in-house breeding colony, and heterozygous genotype was confirmed as previously described [52]. Congenic wildtype (WT) C57BL/6 dams (8 weeks old) were purchased from Charles Rivers Laboratories (Wilmington, MA, USA). Husbandry practices and genotyping were performed as previously described [52]. All animal experiments complied with ARRIVE guidelines.

### 4.2. PCB Dosing

PCB 95 (2,2′,3,5′,6-pentachlorobiphenyl; 99.7% purity; AccuStandard; New Haven, CT, USA) dissolved in organic peanut oil (Spectrum Organic Products, LLC, Melville, NY, USA) was homogeneously mixed into organic peanut butter (Trader Joe’s, Monrovia, CA, USA). After one week of acclimatization to ingestion of peanut butter, dosing was initiated. WT females (see Table 1 for numbers of dams per group) were randomly assigned to one of the following groups using a random number generator (Microsoft Office Excel): vehicle (peanut oil in peanut butter) or PCB 95 at 0.1, 1.0, or 6.0 mg/kg/d. To ensure consistent dosing between animals and across the study, dams were weighed, and the amount of PCB 95-peanut butter mixture was adjusted for body weight. Two weeks after dosing was initiated, females were mated with R163C-*RYR1* heterozygous males randomly assigned using a random number generator (Microsoft Office Excel). Dosing continued daily throughout gestation and lactation up to postnatal day (P) 21, when pups were weaned. During dosing, dams were separated from their litters and placed in a clean cage to ensure that each consumed the entire dose (which typically occurred within 5 min) and that the pups did not have access to the peanut butter. At P2, litters were randomly culled or cross-fostered within dose groups to 7 to 8 pups per litter. When possible, the numbers of males and females were equalized within a litter. The litter served as the experimental unit for all experiments. Within each litter, a maximum of 2 pups per genotype per sex was randomly selected for experimentation—one of these pups was trained, and the other served as an untrained control. For behavioral studies, 9–11 litters per group were used; for non-behavioral endpoints, 5–8 litters per group were used.

### 4.3. Morris Water Maze (MWM)

Weanling mice from each dose group (1 per sex per genotype per litter) were trained in the MWM beginning on P23-P25 as previously described [52]. Untrained controls included littermates that were not trained in the MWM, but that were otherwise similarly handled and housed. Briefly, MWM-trained weanlings were trained for 6 days in blocks of 2 trials/day with an inter-trial interval of approximately 30 min. Trials were conducted at the same time of the day across all experiments and cohorts; water temperature and light levels were kept constant. The order in which animals were tested was varied each day. A probe test, during which the platform was removed from the pool, was administered 24 h after day 6 of training. A visual cue test, in which the platform was made obviously visible above the surface of the water, was conducted approximately 30 min after the probe test. All swim paths were recorded and analyzed using SMART video tracking and analysis software (version 2.5, Panlab, Barcelona, Spain). Spatial learning was assessed as the difference from the beginning to the end of the training period with respect to (i) the time to locate the platform [delta(escape latency)] and (ii) the percentage of the swim path spent in the target quadrant that contained the platform [delta(% pathlength)]. Spatial memory was assessed in the probe test by determining the percentage of swim time (% time) spent in the target quadrant and the number of times the weanlings crossed the former location of the platform (platform crossings). Motivation and sensorimotor function were assessed by determining escape latency and mean swim velocity during the visual cue test [52]. Individuals conducting the behavioral task and analyzing the videotapes were blinded to group.

### 4.4. Morphometric Analysis of Dendritic Arborization

Brains were collected from pups 24 h after completion of MWM testing. MWM-trained pups and their untrained littermate controls (age-, sex-, genotype-, and dose-matched) were euthanized by cervical dislocation. Brains were excised from the skull and bisected into hemispheres, and one hemisphere was immediately immersed in Golgi solution according to the manufacturer’s instructions (FD NeuroTechnologies, Columbia, MD, USA) as previously described [52]. After immersion in solution C, brains were transferred to 10% sucrose in phosphate-buffered saline (PBS; 137 mM NaCl, 10 mM sodium phosphate dibasic, 1.8 mM sodium phosphate dibasic, pH 7.2) for 3–4 h at 4 °C and then held in 30% sucrose in PBS at 4 °C until sectioned. Prior to sectioning, brains were transferred to 70% ethanol in distilled water for 4 h at 4 °C. Brains were cut into 100 µm thick coronal sections in chilled 70% ethanol using a vibratome (VT-1000, Leica, Solms, Germany), and then mounted on gelatin-subbed slides. Brightfield image stacks of CA1 hippocampal pyramidal neurons were captured using an IX-81 inverted microscope (Olympus, Shinjuku, Japan) and analyzed using MetaMorph Image Analysis Software (version 7.1, Molecular Devices, San Jose, CA, USA). Individual neurons were selected for morphometric analyses using previously described criteria [52]. Basilar dendritic arbors of individual Golgi-stained neurons were traced using Neurolucida (version 11, MBF Bioscience, Williston, VT, USA), and the Sholl analysis tool in Neurolucida Explorer software (version 11, MBF Bioscience) was used to quantify dendritic complexity [52]. Six to seven neurons were quantified in each of 5 brains from each experimental group for a total of at least 30 neurons per experimental condition. Individuals conducting the morphometric analyses were blinded to group.

### 4.5. Quantification of PCB 95 in Tissue

PCB 95 and five of its hydroxylated metabolites, 2,2′,4,5′,6-pentachlorobiphenyl-3-ol (3-103), 2,2′,3,5′,6-pentachlorobiphenyl-4-ol (4-95), 2,2′,3,5′,6-pentachlorobiphenyl-4′-ol (4′-95), 2,2′,3,5′,6-pentachlorobiphenyl-5-ol (5-95) and 2,2′,3,5′,6-pentachlorobiphenyl-4,5-diol (4,5-95) were quantified in adipose tissue, liver, brain, and whole blood by gas chromatography with electron capture detection (GC-ECD) as previously described [95] and were adjusted for tissue wet weights to facilitate comparison with earlier studies [50]. To obtain sufficient signal, samples of the same sex, genotype, training status, and dose group were pooled within cohorts.

### 4.6. Thyroid Hormone (TH) and Steroid Hormone (SH) Assays

Serum TH and SH levels were quantified using a flow-based Luminex^®^ 100 suspension array system (Bio-Plex 200, Bio-Rad Laboratories Inc., Hercules, CA, USA). Whole blood (50–200 µL) was collected at the time of euthanasia (24 h after MWM testing) and allowed to clot for at least 30 min. After clotting, samples were spun down at 2500× *g* for 10 min, and then the serum was collected and frozen at −20 °C until extracted. Extraction was performed according to BioRad’s acetonitrile precipitation protocol with the modification that volumes were reduced by a factor of 5 to 15, and samples were reconstituted in 60 µL of assay buffer to accommodate the small amount of blood collected. Circulating levels of the THs triiodothyronine (T3) and thyroxine (T4), and the steroid hormones progesterone, estradiol, and cortisol, were calculated using Bio-Plex Manager software (version 6.0, Bio-Rad Laboratories Inc.) based on a standard curve derived from known reference hormone concentrations supplied by the manufacturer. Final concentrations are expressed as (ng hormone/mL)/mL extracted serum.

### 4.7. Quantitative PCR (qPCR)

Transcript levels of the TH-responsive genes myelin basic protein (MBP) and neurogranin (RC3) and the housekeeping genes hypoxanthine-guanine phosphoribosyl-transferase (HPRT), phosphoglycerate kinase (PGK1), and peptidylprolyl isomerase A (PPIA) were measured in hippocampi, cortices, and cerebella as previously described [52].

### 4.8. Equilibrium [^3^H]Ry Binding Assays of Cortical Homogenates

Cortices were isolated from weanlings 24 h after completion of MWM testing and kept in ice-cold buffer containing 300 mM sucrose, 5 mM imidazole, 100 μM phenylmethanesulfonylfluoride, 10 μg/mL leupeptin, 0.5 mM sodium orthovanadate, 10 mM sodium fluoride, 2 mM β-glycerophosphate, and 5 mM sodium pyrophosphate. The tissue was homogenized in a Teflon–glass homogenizer 3 times for 30 s each, followed by centrifugation at 3500× *g* for 3 min. The supernatant was then centrifuged at 110,000× *g* for 1 h at 4 °C. The pellet was resuspended in 300 mM sucrose and 20 mM HEPES at pH 7.4, flash-frozen in liquid nitrogen, and stored at −80 °C until use. Specific binding of [^3^H]Ry to cortical homogenates was performed as previously described [59] with some modifications. Briefly, protein (500 µg/mL) was incubated for 3 h at 37 °C in binding buffer (250 mM KCl, 15 mM NaCl, 20 mM HEPES, pH 7.4), 5 nM [^3^H]Ry (specific activity of 86.6 µCi/mmole; Perkin Elmer, Waltham, MA, USA), and 200 µM free calcium. Each experiment was performed with 5 data points. Non-specific binding of [^3^H]Ry was determined using samples with a 1000-fold excess of unlabeled ryanodine.

### 4.9. Statistics

All experimental protocols, including the research question, key design features, and analysis plan, were prepared prior to initiation of experimentation; these plans were not registered. No inclusion or exclusion criteria were set for animals used in experimentation, and no animals, experimental units, or data points were excluded from analyses with the exception of the Golgi analyses, for which hippocampi were analyzed only from vehicle control and PCB 95 1.0 mg/kg/d dose groups. Sample size was determined using power calculations based on prior data. Toxicity, PCB 95 and metabolite levels, and Golgi data were assessed using GraphPad Prism (version 10.4.1; GraphPad Software, San Diego, CA, USA). All other data were analyzed using SPSS (version 24, IBM, Armonk, NY, USA). Data were assessed for normality, homogeneity of variance, and sphericity using the Shapiro–Wilk test, Levene’s test, and Mauchly’s test, respectively. Data that were not normally distributed were log-transformed, and the F statistics from data that violated the assumption of sphericity were corrected using the Greenhouse-Geisser correction. Linear mixed-effects models were fit to weight, behavior, TH/SH, and [^3^H]Ry binding data with dose, sex, genotype, training day, or training status as the fixed factors and cohort and individual pup IDs as random factors. Pairwise comparisons were used to identify significant differences between experimental groups (effects of dose, sex, genotype, or training) and between specific experimental conditions (effects of genotype, sex, or training within or between dose groups), with differences considered statistically significant at *p* < 0.05. Toxicity and PCB 95 and metabolite levels were analyzed using one-way ANOVA with a *post hoc* Tukey’s test to identify differences between dose groups. Golgi stain data were analyzed using a linear mixed-effects model fitted by restricted maximum likelihood (REML) with dose, sex, genotype, and training status as the fixed factors and cohort and individual pup IDs as random factors. For qPCR data, C_q_ values for the target genes were normalized to the geometric mean of C_q_ values for the reference genes. Relative expression ratios between experimental groups were statistically analyzed using REST2009 software as previously described [52]. [^3^H]Ry binding data are presented as geometric mean ratios (GMR), which is the ratio of geometric means used to compare continuous outcomes. A GMR of 1.0 indicates no difference between the test group (numerator value) and the comparator group (denominator value).

## Figures and Tables

**Table 1:** Number of dams dosed and litters born in individual cohorts.

Dose	0 mg/kg/day		0.1 mg/kg/day		1.0 mg/kg/day		6.0 mg/kg/day	
Cohort	# Dams	# Litters	# Dams	# Litters	# Dams	# Litters	# Dams	# Litters
1	10	5	10	5	10	4	11	5
2	10	3	10	3	11	3	11	1
3	8	2	7	2	7	1	14	2
4	7	2	6	3	11	2	11	3
5	10	3	4	2	10	3	10	3

## Data Availability

The raw data supporting the conclusions of this article are available in the Dryad repository: DOI: 10.5061/dryad.vmcvdnd4t.

## Supplementary Materials

The following supporting information can be downloaded at https://www.mdpi.com/article/10.3390/ijms27177801/s1.

## Abbreviations

The following abbreviations are used in this manuscript:

MWM	Morris Water Maze
PCB	Polychlorinated Biphenyl
P	Postnatal Day
RYR	Ryanodine Receptor
SH	Steroid Hormone (progesterone, estradiol, and cortisol)
TH	Thyroid Hormone (triiodothyronine, or T3, and thyroxine, or T4)
WT	Wildtype
HET	R163C-*RYR1* Heterozygous
