# Supplementary material for: Expression of the Human R163C-RYR1 Gain-of-Function Mutation Modified 2,2′,3,5′,6-Pentachlorobiphenyl (PCB 95) Developmental Neurotoxicity in Weanling Mice
Source: Int J Mol Sci. 2026 Aug 31;27(17):7801. doi: 10.3390/ijms27177801 (PMC13565785; doi:10.3390/ijms27177801)
Supplement: Supplementary file 1 [file ijms-27-07801-s001.zip › ijms-4498156-supplementary.pdf]

Supplementary Files

R163C-*RYR1* Expression Enhances Hippocampal Dendritic Arborization, Improves Morris Water Maze Performance and Modifies PCB 95 Developmental Neurotoxicity in Weanling Mice

Christopher D Barnhart[1*#], Rebecca J Wilson[1*], Sunjay Sethi[1*], Wei Feng[1], Hans-Joachim Lehmler[2], Isaac N Pessah[1], and Pamela J Lein[1]

[1] Department of Molecular Biosciences, School of Veterinary Medicine, University of California Davis, 1089 Veterinary Medicine Drive, Davis, CA, 95616, USA; cdbarnhart@ucdavis.edu; rjwilso@ucdavis.edu, ssethi@ideayabio.com; inpessah@ucdavis.edu; pjlein@ucdavis.edu

[2] Department of Occupational and Environmental Health, College of Public Health, University of Iowa, Iowa City, Iowa 52242, United States

* Co-first author

\# Currently employed by the National Institute of Health Tribal Health Research Office

Correspondence to Pamela J. Lein: pjlein@ucdavis.edu; Tel (530) 752-1970

Supplementary Table S1. Statistical analysis of PCB 95 exposure, training, sex, and R163C-*RYR1* expression on circulating steroid hormones in P31-33 weanling mice.

Variables		Cortisol		Estradiol (Log)		Progesterone (Log)	
	Dose Comparison	F Statistic	P Value	F Statistic	P Value	F Statistic	P Value
Dose Group	0.1 mg/kg/d	F(1,73.889) = 0.186	p=0.668	F(1,72.88) = 0.348	p=0.557	F(1,72.767) = 1.259	p=0.266
	1.0 mg/kg/d	F(1,69.789) = 1.057	p=0.308	F(1,65.033) = 0.447	p=0.506	F(1,69.110) = 3.155	p=0.080
	6.0 mg/kg/d	F(1,74.915) = 0.619	p=0.434	F(1,68.820) = 0.003	p=0.957	F(1,73.981) = 0.575	p=0.451
Training	0.1 mg/kg/d	**F(1,74.404) = 5.208**	**p=0.025**	F(1,71.941) = 3.077	p=0.084	**F(1,73.136) = 5.009**	**p=0.028**
	1.0 mg/kg/d	**F(1,69.589) = 8.089**	**p=0.006**	F(1,64.643) = 1.482	p=0.228	F(1,68.759) = 1.507	p=0.224
	6.0 mg/kg/d	**F(1,71.867) = 6.096**	**p=0.016**	F(1, 68.442) = 2.394	p=0.126	F(1,70.953) = 2.377	p=0.128
Sex	0.1 mg/kg/d	**F(1,73.287) = 30.301**	**p=0.000**	F(1,70.608) = 0.065	p=0.800	F(1,72.022) = 4.833	p = 0.031
	1.0 mg/kg/d	**F(1,69.594) = 23.727**	**p=0.000**	F(1,64.736) = 0.037	p=0.849	F(1,68.730) = 3.000	p=0.088
	6.0 mg/kg/d	**F(1,73.634) = 14.135**	**p=0.000**	F(1,68.862) = 0.942	p=0.335	**F(1,72.820) = 12.295**	**p= 0.001**
Genotype	0.1 mg/kg/d	F(1,75.061) = 1.817	p=0.182	F(1,72.943) = 0.593	p=0.444	F(1,73.825) = 1.358	p = 0.248
	1.0 mg/kg/d	F(1,70.357) = 0.012	p=0.914	F(1,65.863) = 0.224	p=0.638	F(1, 69.807) = 0.379	p=0.540
	6.0 mg/kg/d	F(1,73.112) = 0.189	p=0.665	F(1,69.076) = 0.624	p=0.432	F(1, 72.172) = 0.282	p=0.597

Values in the table represent F values, including degrees of freedom, and the corresponding p-values following pairwise comparisons. Bolded cells indicate $p < 0.05$ (n=4-6 animals per experimental condition).

Supplementary Table S2. **Statistical analysis of PCB 95 exposure, training, sex, and R163C-*RYR1* expression on circulating thyroid hormones in P31-33 weanling mice.**

Variables		T3 (Log)		T4	
	Dose Comparison	F Statistic	P Value	F Statistic	P Value
Dose Group	0.1 mg/kg/d	F(1,75.443) = 0.092	p=0.762	F(1,77) = 1.957	p=0.166
	1.0 mg/kg/d	F(1,69.957) = 0.040	p=0.842	F(1,71.630) = 1.862	p=0.177
	6.0 mg/kg/d	F(1, 74.976) = 0.001	p=0.978	F(1,75) = 0.221	p=0.639
Training	0.1 mg/kg/d	**F(1,75.235) = 4.966**	**p = 0.029**	F(1,77) = 2.132	p=0.148
	1.0 mg/kg/d	F(1,69.569) = 1.631	p=0.206	F(1,70.494) = 0.109	p=0.742
	6.0 mg/kg/d	F(1,71.824) = 1.469	p=0.229	F(1,75) = 0.652	p=0.422
Sex	0.1 mg/kg/d	F(1, 74.256) = 3.011	p=0.087	**F(1,77) = 10.180**	**p=0.002**
	1.0 mg/kg/d	F(1, 69.610) = 2.122	p=0.150	F(1,70.712) = 1.971	p=0.165
	6.0 mg/kg/d	F(1,73.814) = 0.085	p=0.772	F(1,75) = 1.611	p=0.208
Genotype	0.1 mg/kg/d	F(1,76.440) = 0.403	p=0.527	F(1,77) = 0.482	p=0.489
	1.0 mg/kg/d	F(1,71.093) = 0.346	p=0.558	F(1, 72.225) = 2.201	p=0.142
	6.0 mg/kg/d	F(1,73.398) = 0.171	p=0.681	F(1,75) = 1.257	p=0.266

T3=Triiodothyronine; T4 = Thyroxine. Values in the table represent F values, including degrees of freedom, and the corresponding p-values following pairwise comparisons. Bolded cells indicate $p < 0.05$ (n=4-6 animals per experimental condition).

Supplementary Table S3. Effects of R163C-*RYR1* expression on TH-responsive gene expression in untrained (UNT) and MWM-trained weanlings.

	0 mg/kg/day			0.1 mg/kg/day			1 mg/kg/day			6 mg/kg/day		
**Comparison**	CX	HC	CB	CX	HC	CB	CX	HC	CB	CX	HC	CB
UNT WT v HET												
MBP	0.78	1.11	0.98	1.11	1.18	1.32	0.85	1.02	1.00	1.29	0.96	0.79
RC3	0.55	0.85	1.24	1.25	1.17	1.48	0.73	1.11	0.49	1.00	1.12	1.13
MWM WT v HET												
MBP	1.04	0.72	1.27	1.01	1.21	1.02	0.94	1.00	1.26	0.64	0.89	0.55
RC3	1.01	1.50	1.20	0.95	1.12	0.58	1.05	1.09	1.57	0.69	**0.80**# (95%: 0.39-1.29)	0.71

CX = cortex, HC = hippocampus, CB = cerebellum, WT = wild type weanlings, HET = R163C-*RYR1* heterozygous weanlings, MBP = myelin basic protein, RC3 = neurogranin. Values in the tables represent fold-change in transcript levels. #$p < 0.05$ as determined by REST2009 analysis with the indicated 95% confidence interval (n = 8-12 animals per experimental condition).

Supplementary Table S4. Effects of PCB 95 dose on TH-responsive gene expression in untrained (UNT) and MWM-trained weanlings.

	0 v 0.1 mg/kg/day			0 v 1 mg/kg/day			0 v 6 mg/kg/day		
**Comparison**	CX	HC	CB	CX	HC	CB	CX	HC	CB
UNT WT									
MBP	1.05	0.99	0.79	1.08	1.33	1.22	0.76	1.12	1.11
RC3	0.74	0.82	0.83	0.87	0.86	1.77	0.74	0.79	1.00
UNT HET									
MBP	1.51	1.03	1.06	1.17	1.20	1.24	1.26	0.94	0.89
RC3	1.39	1.11	1.31	1.15	1.09	0.66	1.34	1.02	1.04
MWM WT									
MBP	0.98	0.97	0.94	1.06	0.87	0.89	0.98	0.94	1.36
RC3	0.83	0.98	0.84	1.01	1.00	0.45	1.05	1.08	0.60
MWM HET									
MBP	0.95	**1.63**# (95%: 0.61-7.16)	0.76	0.96	1.21	0.89	0.61	1.16	0.58
RC3	0.77	1.05	0.44	1.03	1.15	0.58	0.70	0.91	0.37

CX = cortex, HC = hippocampus, CB = cerebellum, WT = wild type weanlings, HET = R163C-*RYR1* heterozygous weanlings, MBP = myelin basic protein, RC3 = neurogranin. Values in the tables represent fold-change in transcript levels. #$p < 0.05$ as determined by REST2009 analysis with the indicated 95% confidence interval (n = 8-12 animals per experimental condition).

Supplementary Table S5. Effects of MWM training on TH-responsive gene expression in WT and HET weanlings.

	**0 mg/kg/day**			**0.1 mg/kg/day**			**1 mg/kg/day**			**6 mg/kg/day**		
**Comparison**	CX	HC	CB	CX	HC	CB	CX	HC	CB	CX	HC	CB
WT UNT v MWM												
MBP												
RC3	0.94	1.28	1.00	0.88	1.26	1.20	0.93	0.84	0.73	1.23	1.08	1.23
	0.77	0.85	2.15	0.86	0.91	1.74	0.89	0.98	0.54	1.09	1.15	1.02
HET UNT v MWM												
MBP												
RC3	1.26	0.81	1.23	0.79	1.29	0.92	1.04	0.82	0.93	0.61	1.00	0.86
	1.43	0.92	1.28	0.65	0.87	0.92	1.28	0.97	1.67	0.75	0.82	0.66

CX = cortex, HC = hippocampus, CB = cerebellum, WT = wild type weanlings, HET = R163C-*RYR1* heterozygous weanlings, MBP = myelin basic protein, RC3 = neurogranin. Values in the tables represent fold-change in transcript levels (n = 8-12 animals per experimental condition).

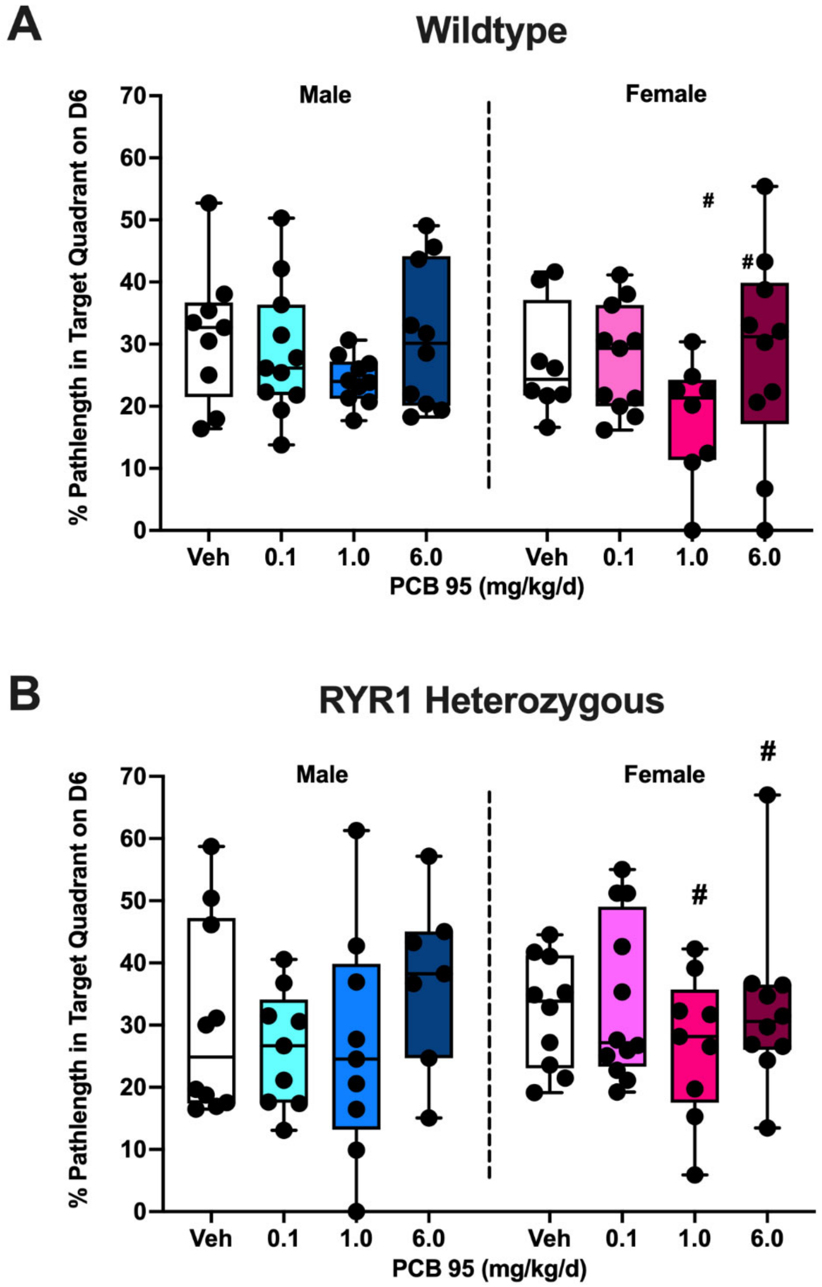

Supplementary Figure S1. Effects of PCB 95 and R163C-RyR1 expression on percent of pathlength spent in target quadrant. On the 6th, and final, day of Morris Water Maze training, (A) WT and (B) HET weanlings were assessed for the percent of total swim pathlength that located in the target quadrant. Data are presented as box and whisker plots in which the box indicates the 25th (lower) to 75th (upper) quartiles; the middle horizontal bar, the median; whiskers, the minimum and maximum values; and dots, the individual values (n = *9-11* animals per group). # Significantly different from the dose- and sex- matched WT control group at $p < 0.05$, as determined by linear mixed-effects model analysis.

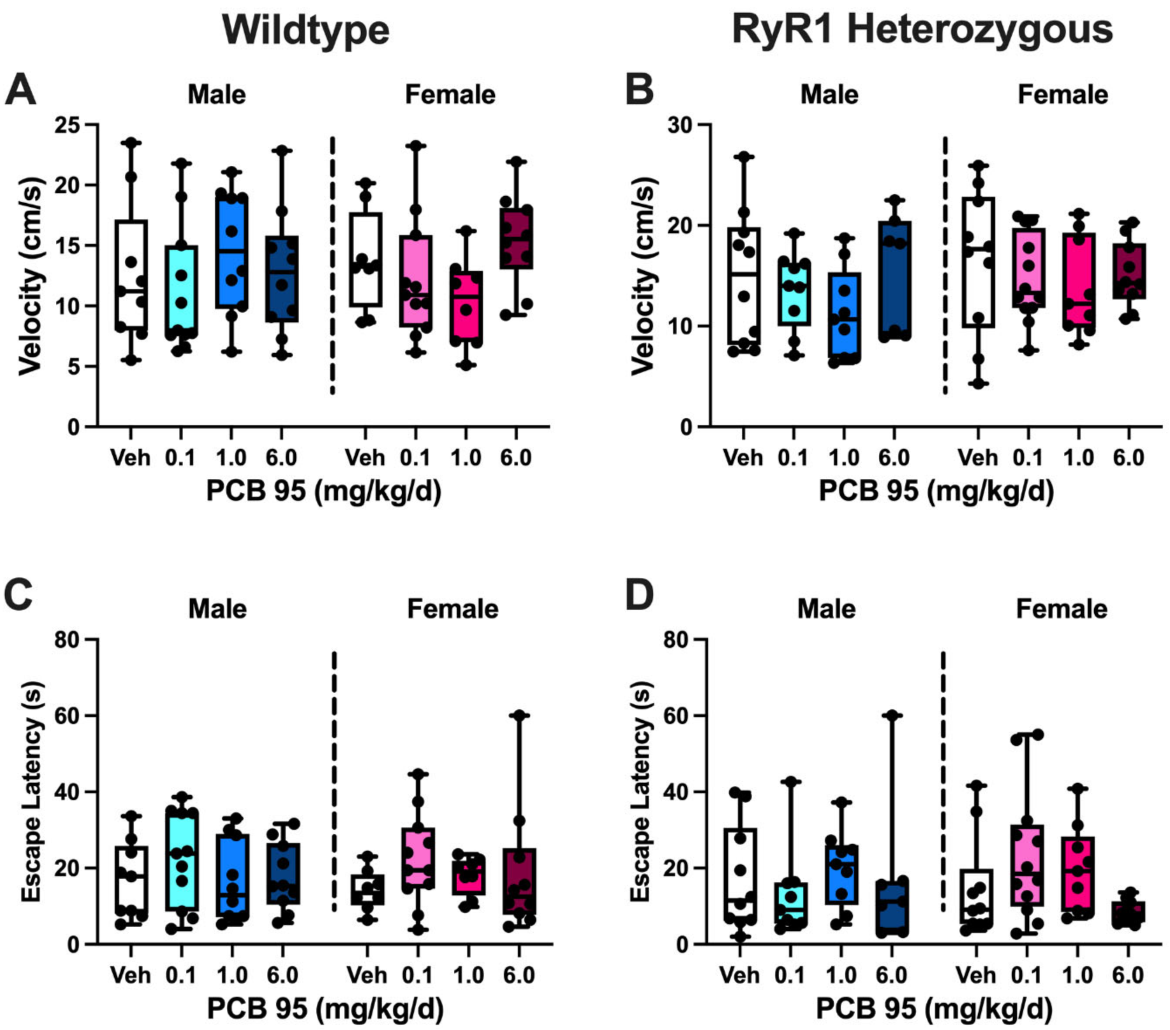

Supplementary Figure S2. PCB 95 and R163C-RyR1 expression had no effect on performance in the visual cue task of the Morris Water Maze. Following the probe test of Morris Water Maze training, weanlings were given a visual cue task to rule out deficits in visual and sensorimotor function. (A-B) Mean swim velocity and (C-D) escape latency were measured in (A,C) wildtype and (B,D) HET weanlings. Data are presented as box and whisker plots in which the box indicates the 25th (lower) to 75th (upper) quartiles; the middle horizontal bar, the median; whiskers, the minimum and maximum values; and dots, the individual values (n = *9-11* animals per group).

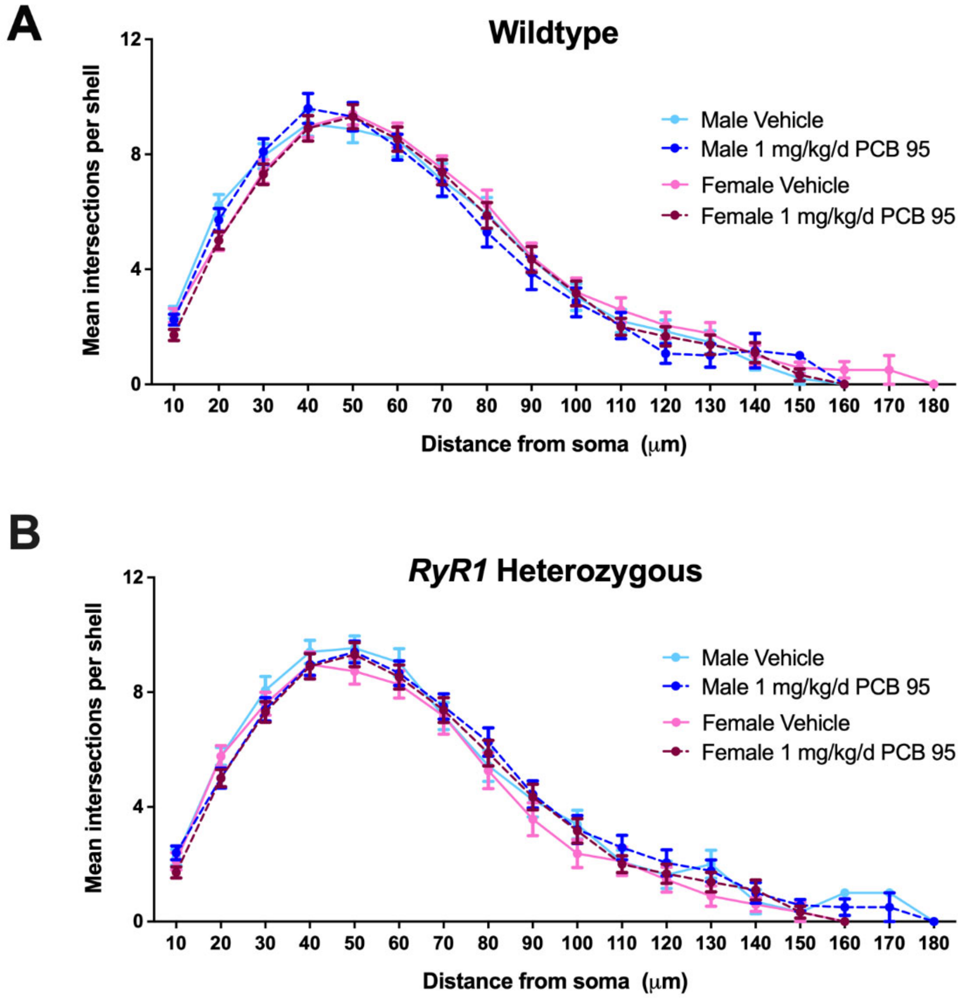

Supplementary Figure S3. WT and HET weanlings showed no significant sex differences in dendritic arborization. Sholl plots generated from Sholl analysis of the basilar arbors of CA1 pyramidal neurons at P31-33 in (A) WT and (B) HET weanlings exposed to vehicle vs. PCB 95 at 1 mg/kg/d in the maternal diet. Data are presented as the mean ± SEM ($n \geq 30$ neurons from at least 5 animals per experimental condition).
